# Genome-wide profile analysis of the Hsp20 family in lettuce and identification of its response to drought stress

**DOI:** 10.3389/fpls.2024.1426719

**Published:** 2024-07-12

**Authors:** Qinqin Zhang, Bowen Dai, Mi Fan, Liling Yang, Chang Li, Guangguang Hou, Xiaofang Wang, Hongbo Gao, Jingrui Li

**Affiliations:** ^1^ College of Horticulture, Hebei Agricultural University, Baoding, China; ^2^ Key Laboratory of North China Water-saving Irrigation Engineering, Hebei Agricultural University, Baoding, China; ^3^ Ministry of Education of China-Hebei Province Joint Innovation Center for Efficient Green Vegetable Industry, Baoding, China

**Keywords:** gene family, lettuce, LsHsp20, drought, gene expression

## Abstract

Heat shock protein 20 (Hsp20) plays a very important role in response to abiotic stressors such as drought; however, in lettuce (*Lactuca sativa* L.), this gene family is poorly understood. This study used bioinformatics methods to identify 36 members of the lettuce Hsp20 family, which were named *LsHsp20–1~LsHsp20–36*. Subcellular localization results revealed that 26 members of the LsHsp20 protein family localized to the cytoplasm and nucleus. Additionally, 15 conserved domains were identified in the LsHsp20 protein family, with the number of amino acids ranging from 8 to 50. Gene structure analysis revealed that 15 genes (41.7%) had no introns, and 20 genes (55.5%) had one intron. The proportion of the *LsHsp20* secondary structure was random coil > alpha helix > extended strand > beta turn. Chromosome positioning analysis indicated that 36 genes were unevenly distributed on nine chromosomes, and four pairs of genes were collinear. The Ka/Ks ratio of the collinear genes was less than 1, indicating that purifying selection dominated during *L*. *sativa* evolution. Thirteen pairs of genes were collinear in lettuce and *Arabidopsis*, and 14 pairs of genes were collinear in lettuce and tomato. A total of 36 LsHsp20 proteins were divided into 12 subgroups based on phylogenetic analysis. Three types of cis-acting elements, namely, abiotic and biotic stress-responsive, plant hormone-responsive, and plant development-related elements, were identified in the lettuce *LsHsp20* family. qRT-PCR was used to analyze the expression levels of 23 *LsHsp20* genes that were significantly upregulated on the 7th or 14th day of drought treatment, and the expression levels of two genes (*LsHsp20–12* and *LsHsp20–26*) were significantly increased by 153-fold and 273-fold on the 14th and 7th days of drought treatment, respectively. The results of this study provide comprehensive information for research on the LsHsp20 gene family in lettuce and lay a solid foundation for further elucidation of *Hsp20* biological functions, providing valuable information on the regulatory mechanisms of the LsHsp20 family in lettuce drought resistance.

## 1 Introduction

Heat shock proteins (Hsps) are an evolutionarily ancient, highly conserved group of intracellular molecules that are present in the cells of almost all organisms, including archaea, prokaryotes, and eukaryotes (Vierling, 1991; Waters, 2013). Heat shock proteins are responsible for assisting in the correct folding, assembly, and transport of proteins. They can maintain the conformational and functional stability of proteins by rebuilding normal proteins under stress conditions, participating in other stress response mechanisms, and playing an important role in protecting plants from abiotic stress (Wang et al., 2004). Research shows that when plants are subjected to various stresses, the Hsp protein content in the plant rapidly increases to help the plant resist stress (Zhang et al., 2015; Haq et al., 2019; Huang et al., 2022).

Ritossa first discovered heat shock protein in *Drosophila* salivary glands (Ritossa, 1962). Vierling was the first to discover Hsps in soybean plants, after which Nover et al. treated tomato cells at high temperatures and identified similar proteins related to high-temperature stress (Nover et al., 1983; Vierling, 1991). The functions of Hsps are also gradually being explored. Many studies have shown that Hsp expression is regulated by high temperatures as well as by abiotic stresses such as drought. Hsps can be divided into five categories according to their amino acid sequence homology and molecular weight: the high-molecular-weight protein families Hsp100, Hsp90, Hsp70, and Hsp60 and the low-molecular-weight protein family Hsp20. Hsp20 proteins are also called small heat shock proteins, and their molecular weights are between 15 and 42 kDa (Chen et al., 2018b; Zhao et al., 2018). The structure of the family of Hsp20 proteins includes an exogenous signal receiving end, an N-terminal variable domain, a C-terminal conserved domain, and an a-crystalline protein domain, which is also called ACD (Bondino et al., 2012; Lin et al., 2019). Moreover, these three regions play different roles: the N-terminal region is involved in regulating substrate binding and oligomerization, the C-terminal extension contains an amino acid motif that promotes homo-oligomerization and maintains organelle specificity, and the ACD has a conserved β-sheet structure and is composed of 80 to 100 amino acids that interact with the substrate (Waters, 2013; Peng et al., 2023). Hsp20 proteins have the largest number of members in plants (Hu et al., 2021). Hsp20 proteins are ATP-independent molecular chaperones with a strong ability to bind to denatured substrates and can maintain protein stability, prevent proteins from irreversibly aggregating or denaturing, and promote protein transport through membrane channels, thus preventing damage to proteins caused by abiotic stress (Haslbeck and Vierling, 2015). Therefore, Hsp20 proteins play an important role in improving plant tolerance and protecting plants from stress.

Since the discovery of the importance of *Hsp20* genes in coping with various biotic and abiotic stresses, Hsp20 gene family members have been identified in various plants; for example, 30, 48, 42, 38, and 42 Hsp20 gene family members have been identified in *Arabidopsis* (Huang et al., 2023), potato (Zhao et al., 2018), tomato (Yu et al., 2016), barley (Li and Liu, 2019), and peach (Lian et al., 2022), with 14, 12, 13, 7 and 11 subgroups, respectively. Studies have shown that *Hsp17.6B* overexpression in *Arabidopsis* significantly increases root elongation, plant survival rate, electrolyte leakage rate, and chlorophyll content under heat stress (Tawab et al., 2019). The overexpression of *CaHsp16.4* in pepper plants enhances the scavenging of reactive oxygen species produced under stress, leading to enhanced heat tolerance and drought resistance (Huang et al., 2019). In tomatoes, *Hsp20* genes can respond to high temperature, drought, and high salt stress to varying degrees. *Hsp20* genes are also involved in tomato fruit development. It is more actively expressed during the fruit maturity period than during other periods, and its expression increases as the fruit matures (Yu et al., 2016). Studies in peach plants have shown that overexpression of the *PpHsp20–32* gene is involved in the regulation of plant height and enhancing heat tolerance (Lian et al., 2022). The overexpression of *FaHsp17.4* in strawberry plants is also involved in the regulation of strawberry fruit growth and development (Li et al., 2016). The *Hsp20* genes in *Coix* can respond to high temperatures and drought to varying degrees and regulate growth and development (Hua et al., 2023). Taken together, these results indicate that the Hsp20 family plays an important positive role in improving plant immunity and alleviating abiotic stress.

Lettuce (*Lactuca sativa* L.) is one of the most widely planted vegetable species worldwide. Due to its shallow root distribution, poor water absorption capacity, and large water demand throughout its growth period, lettuce has high soil moisture requirements, and drought stress is one of the main factors affecting its growth and yield (Chen et al., 2018a; Balko et al., 2023; Ceylan et al., 2023). The lettuce Hsp20 gene family has not been systematically studied among the species in which it has been identified. Therefore, this study was based on lettuce genome information and used bioinformatics methods to identify members of the lettuce Hsp20 gene family and analyze their physical and chemical properties, conserved domains, and chromosomal locations. qRT-PCR technology was used to analyze the expression patterns of members of this gene family under drought stress, providing basic information for in-depth research on the function of the Hsp20 gene family in vegetables and its role in responding to adverse stress.

## 2 Materials and methods

### 2.1 Identification of LsHsp20 family members in lettuce

The lettuce reference genome and protein sequences were obtained from the Ensembl Plants database (https://plants.ensembl.org/index.html). The *Arabidopsis thaliana* Hsp20 protein family sequence was obtained from the TAIR database (https://www.arabidopsis.org). Hidden Markov model (HMM) files were obtained from the Pfam (PF00011) protein family database (http://pfam-legacy.xfam.org/). The *Arabidopsis* Hsp20 protein sequence was compared with the lettuce genome-wide protein sequence using the TBtools Blast function, and the resulting gene ID was determined in duplicate. To ensure that all candidate Hsp20 members contain ACD domains, the CDD (https://www.ncbi.nlm.nih.gov/structure/bwrpsb/bwrpsb.CGI), Pfam (http://pfam-legacy.xfam.org/), and SMART (http://smart.embl-Heidelberg.de/) databases were used. The relative molecular weights of all candidate members were further screened and predicted, and the Hsp20 members without an ACD domain and with relative molecular weights greater than 15∼42 kDa were removed. After identification and screening, the lettuce Hsp20 family members were obtained, and the genes were named *LsHsp20–1* to *LsHsp20–36* (**Supplementary Tables S1**, **S2**).

### 2.2 Basic information on the LsHsp20 gene family in lettuce

The ExPASy ProtParam (http://cn.expasy.org) program was used to analyze the molecular weights, theoretical isoelectric points, instability coefficients, and hydrophilicity indices of the proteins encoded by the lettuce LsHsp20 gene family. Furthermore, the online tool PSORT (https://wolfpsort.hgc.jp) was used to predict the subcellular localization of the Hsp20 proteins in lettuce.

### 2.3 Analysis of the structure and domain of the *LsHsp20* genes in lettuce

The exon and intron data for the lettuce *LsHsp20* genes were obtained from the database (http://cucurbitgenomics.org), and conserved protein motifs were analyzed with the MEME tool (http://meme.nbcr.net/meme). The maximum motif was set to 15, and the remaining parameters were set to the default values.

### 2.4 Secondary structure and three-dimensional structural model of the lettuce LsHsp20 proteins

The SOPMA (http://npsa-pbil.ibcp.fr) and SWISS-MODEL (https://swissmodel.expasy.org) online tools were used to determine the secondary structures and three-dimensional structural models of the lettuce LsHsp20 proteins.

### 2.5 Analysis of the chromosomal locations, collinearity, and evolutionary selection pressure of the lettuce *LsHsp20* genes

Chromosomal location information for the *LsHsp20* genes was obtained from the Ensembl Plants database (http://plants.bl.org/index.html), and the online tool MG2C V2.1 (http://mg2c.iask.in/mg2c_v2.1) was used to determine the chromosomal locations of the *Hsp20* genes. For the collinearity analysis of the Hsp20 gene family members, the TBtool software and its advanced Circos and Dual synteny plotter functions were used to perform intraspecies and interspecies collinearity analyses on the identified sequences, respectively.

### 2.6 Phylogenetic analysis and classification of the LsHsp20 family in lettuce

The Muscle function in MEGA 7.0 was used to perform multiple sequence alignment of Hsp20 proteins from five species: lettuce, *Arabidopsis* (Huang et al., 2023), tomato (Yu et al., 2016), rice (Ouyang et al., 2009), and barley (Li and Liu, 2019). After the alignment was completed, TBtools was used to trim the sequences. MEGA 7.0 maximum likelihood (ML) was used to construct a phylogenetic tree for lettuce, *Arabidopsis*, tomato, rice, and barley. The bootstrap value was set to 1,000, the gap was set to “pairwise deletion,” and the “Poisson model” was used to verify that the tree was reliable. Based on the subcellular localization prediction for the LsHsp20 family in lettuce, the classification of Hsp20 proteins in other species, and the evolutionary structure of the phylogenetic tree, the LsHsp20 proteins were divided into different subgroups.

### 2.7 Analysis of cis-acting elements in the LsHsp20 gene family

The online prediction tool PlantCARE (http://bioinformatics.psb.ugent.be/webtools/plantcare/html) was used to predict and analyze the 2,000-bp upstream sequence and main cis-acting elements of the lettuce *LsHsp20* genes. The cis-elements in the promoters of *LsHsp20* genes were also predicted.

### 2.8 Plant materials and drought stress treatment

The lettuce cultivar ‘Yidali 151’ (from Beijing Shuoyuan Seed Co., Ltd., Beijing, China) served as the plant material in this study and was cultured in an artificial climate chamber at Hebei Agricultural University. Seeds of full and consistent sizes were selected, a 72-hole plug tray was used for seedling cultivation using coconut peat:vermiculite:perlite at a ratio of 3:1:1 as the substrate, and the seeds were subjected to conventional seedling management at an indoor temperature of 25°C ± 2°C, humidity of 40%, and light intensity of 130 μmol·m^−2^·s^−1^. When the seedlings had six to seven true leaves, they were transplanted into the greenhouse. Drip lateral lines of 16 mm diameter were laid between the two rows, and when the seedlings survived, drought treatment was performed. The soil moisture content was monitored daily by a ZL6 data collector (METER Group, Inc., USA), which was inserted into a depth of 15 cm. The dripper discharge was 1.38 lph at a pressure of 0.1 MPa. When the soil moisture content was approximately 85%–95%, the sample was treated for 0 days (control) and irrigation was stopped, and when the substrate moisture content dropped to 60%–65%, which was marked as treatment, the moisture content of the substrate was maintained within this range. Samples were collected after 7 days, 14 days, and 21 days of drought treatment.

### 2.9 RNA extraction, cDNA synthesis, and real-time fluorescence quantitative PCR

Leaves from the normal and drought-treated lettuce groups were removed for RNA extraction and qRT-PCR analysis. Total RNA was extracted using an EasyPure^®^ RNA Kit (TransGen, Beijing, China). A FastKing cDNA First-Strand Synthesis Kit (Tiangen, Beijing, China) was used to reverse transcribe total RNA to obtain cDNA. The NCBI database was used to design primers for 29 *LsHsp20* genes, and primer information was obtained (**Supplementary Table S3**). The serial number of the reference gene was *LSAT_8X116260*. Real-time fluorescence quantitative PCR was performed using the TransStart Top Green qPCR SuperMix kit (US Everbright, Suzhou, China).

### 2.10 Data analysis

SPSS·27.0 software was used for statistical analysis, and GraphPad Prism 8 software was used for mapping. The asterisks indicate the level of significance (* means *p* < 0.05, ** means *p* < 0.01) based on Duncan’s multiple range test.

## 3 Results

### 3.1 Whole-genome identification and physical and chemical property analyses of the lettuce LsHsp20 family members

The presence of the ACD domain was confirmed via HMM by submitting the protein sequences to the CDD, Pfam, and SMART databases. After deleting sequences without typical ACD domains and sequences with molecular weights exceeding 15 to 42 kDa, 36 LsHsp20 family members were identified in the full lettuce genome, and their physical and chemical properties were analyzed (**Table 1**). The number of amino acids in the LsHsp20 proteins ranged from 137 (LsHsp20–16) to 331 (LsHsp20–18). The molecular weights of LsHsp20 proteins ranged from 15.62 kDa (LsHsp20–16) to 37.62 kDa (LsHsp20–18). The predicted p*I* of LsHsp20 proteins ranged from 4.94 (LsHsp20–6, LsHsp20–25) to 9.49 (LsHsp20–24), the instability index ranged from 24.34 (LsHsp20–27) to 70.9 (LsHsp20–35), the lipophilic index ranged from 62.86 (LsHsp20–19) to 92.65 (LsHsp20–2), and the overall average hydrophobicity index ranged from −0.865 (LsHsp20–3) to −0.207 (LsHsp20–16). Upon assessing subcellular localization, 26 LsHsp20 proteins were localized in the cytoplasm and nucleus, six were localized in chloroplasts, two were localized in the Golgi apparatus, one was localized in the peroxisome, and one was in the endoplasmic reticulum.

**Table 1:** Physicochemical properties of the LsHsp20 proteins.

Gene	Gene ID	Number of amino acids	Molecular weight	Theoretical p*I*	Instability index	Aliphatic index	Grand average of hydropathicity (GRAVY)	Subcellular location
*LsHsp20–1*	LSAT_5X168220	156	17,890.26	5.99	49.51	73.65	−0.694	Cytoplasm
*LsHsp20–2*	LSAT_8X130841	204	23,180.38	5.26	43.65	92.65	−0.49	Chloroplast
*LsHsp20–3*	LSAT_1X33281	211	24,441.67	7.89	50.11	68.82	−0.865	Nucleus
*LsHsp20–4*	LSAT_8X151001	235	26,595.23	8.95	39.23	67.19	−0.719	Chloroplast
*LsHsp20–5*	LSAT_5X171121	154	17,422.78	6.19	52.16	73.96	−0.606	Cytoplasm
*LsHsp20–6*	LSAT_4X107421	236	26,800.84	4.94	60.06	72.67	−0.828	Golgi apparatus
*LsHsp20–7*	LSAT_6X100581	242	27,788.84	6.13	45.6	81.65	−0.638	Cytoplasm
*LsHsp20–8*	LSAT_7X61061	164	18,279.92	6.1	36.32	80.18	−0.493	Cytoplasm
*LsHsp20–9*	LSAT_7X61001	163	18,371.88	6.44	33.18	74.11	−0.634	Cytoplasm
*LsHsp20–10*	LSAT_3X481	215	24,376.36	5.48	53.68	72.93	−0.728	Chloroplast
*LsHsp20–11*	LSAT_5X136741	161	17,777.49	6.84	60.24	88.88	−0.414	Nucleus
*LsHsp20–12*	LSAT_7X60201	163	18,433.05	5.97	34.53	77.12	−0.576	Cytoplasm
*LsHsp20–13*	LSAT_7X108120	191	21,987.17	5.22	53.15	74.4	−0.386	Nucleus
*LsHsp20–14*	LSAT_9X81920	192	21,836.36	7.08	46.42	90.94	−0.482	Cytoplasm
*LsHsp20–15*	LSAT_7X60240	179	19,831.73	5.98	46.13	78.94	−0.522	Cytoplasm
*LsHsp20–16*	LSAT_7X17661	137	15,623.71	5.12	52.59	73.94	−0.207	Cytoplasm
*LsHsp20–17*	LSAT_7X61041	158	17,706.12	6.84	36.1	71.58	−0.642	Cytoplasm
*LsHsp20–18*	LSAT_9X82001	331	37,618.06	8.63	49.18	92.24	−0.319	Endoplasmic reticulum
*LsHsp20–19*	LSAT_2X28080	206	23,699.52	5.67	58.16	62.86	−0.8	Cytoplasm
*LsHsp20–20*	LSAT_2X28120	157	17,985.33	6.01	58.8	70.7	−0.735	Cytoplasm
*LsHsp20–21*	LSAT_2X120081	163	18,499.74	6.33	39.25	66.87	−0.663	Cytoplasm
*LsHsp20–22*	LSAT_2X120060	157	17,851.17	6.01	59.31	75.73	−0.687	Cytoplasm
*LsHsp20–23*	LSAT_1X25141	217	24,832.00	9.43	41.78	83.46	−0.4	Chloroplast
*LsHsp20–24*	LSAT_7X14200	264	29,265.61	9.49	42.87	78.6	−0.617	Cytoplasm
*LsHsp20–25*	LSAT_2X28000	210	23,811.85	4.94	56.19	74.24	−0.64	Cytoplasm
*LsHsp20–26*	LSAT_4X63061	223	25,019.76	8.84	37.61	79.91	−0.603	Cytoplasm
*LsHsp20–27*	LSAT_2X7120	198	21,114.95	5.17	24.34	80.66	−0.249	Cytoplasm
*LsHsp20–28*	LSAT_3X65081	141	15,645.93	6.85	31.12	86.17	−0.345	Peroxisome
*LsHsp20–29*	LSAT_1X56241	188	21,175.22	9.25	32.81	81.33	−0.424	Cytoplasm
*LsHsp20–30*	LSAT_7X61081	163	18,229.89	6.43	40.74	83.68	−0.49	Cytoplasm
*LsHsp20–31*	LSAT_6X91340	215	24,564.05	5.56	61.23	67.53	−0.629	Chloroplast
*LsHsp20–32*	LSAT_8X4541	233	26,148.58	7.61	48.7	71.5	−0.65	Chloroplast
*LsHsp20–33*	LSAT_8X72460	155	17,648.97	5.8	44.51	68.45	−0.693	Cytoplasm
*LsHsp20–34*	LSAT_8X72420	155	17,725.03	5.81	46.91	68.45	−0.715	Cytoplasm
*LsHsp20–35*	LSAT_4X59740	140	16,341.56	4.95	70.9	82.14	−0.321	Cytoplasm
*LsHsp20–36*	LSAT_9X40940	186	20,924.85	5.88	42.3	87.9	−0.511	Golgi apparatus

### 3.2 Analysis of the structures and domains of the *LsHsp20* genes in lettuce

**Figure 1:**
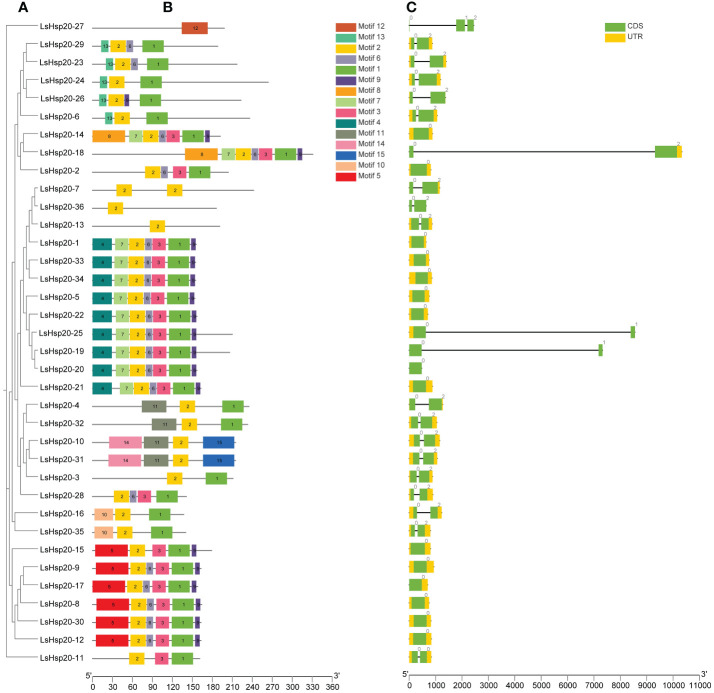
Evolutionary tree **(A)**, motif analysis **(B)**, and gene structure **(C)** of the lettuce LsHsp20 family.

By analyzing the conserved structural domains of the lettuce LsHsp20 family members (**Figure 1A**), a total of 15 conserved domains were identified, with the number of amino acids ranging from 8 to 50 (**Supplementary Table S4**). Among the identified motifs, motif 9 was 8 amino acids wide, while motifs 5, 8, and 14 had widths of 50 amino acids. The number of conserved domains per LsHsp20 protein ranged from 1 to 7. Most of the LsHsp20 contained three to seven conserved domains, while LsHsp20–13, LsHsp20–27, and LsHsp20–36 each contained one conserved domain. Motif 1 (83.3%) and motif 2 (97.2%) appeared more frequently among the lettuce LsHsp20 family members (**Figure 1B**). Based on the results of the Pfam and SMART analyses, motifs 1 and 2 are ACD structures and may play important roles in the stress response in lettuce. An analysis of the gene structure of the 36 identified lettuce LsHsp20 gene family members revealed that among the *LsHsp20* genes, 41.7% had no introns, 55.6% had one intron, and 2.7% had two introns (**Figure 1C**).

### 3.3 Secondary structure and three-dimensional structural model of the lettuce LsHsp20 protein family

**Figure 2:**
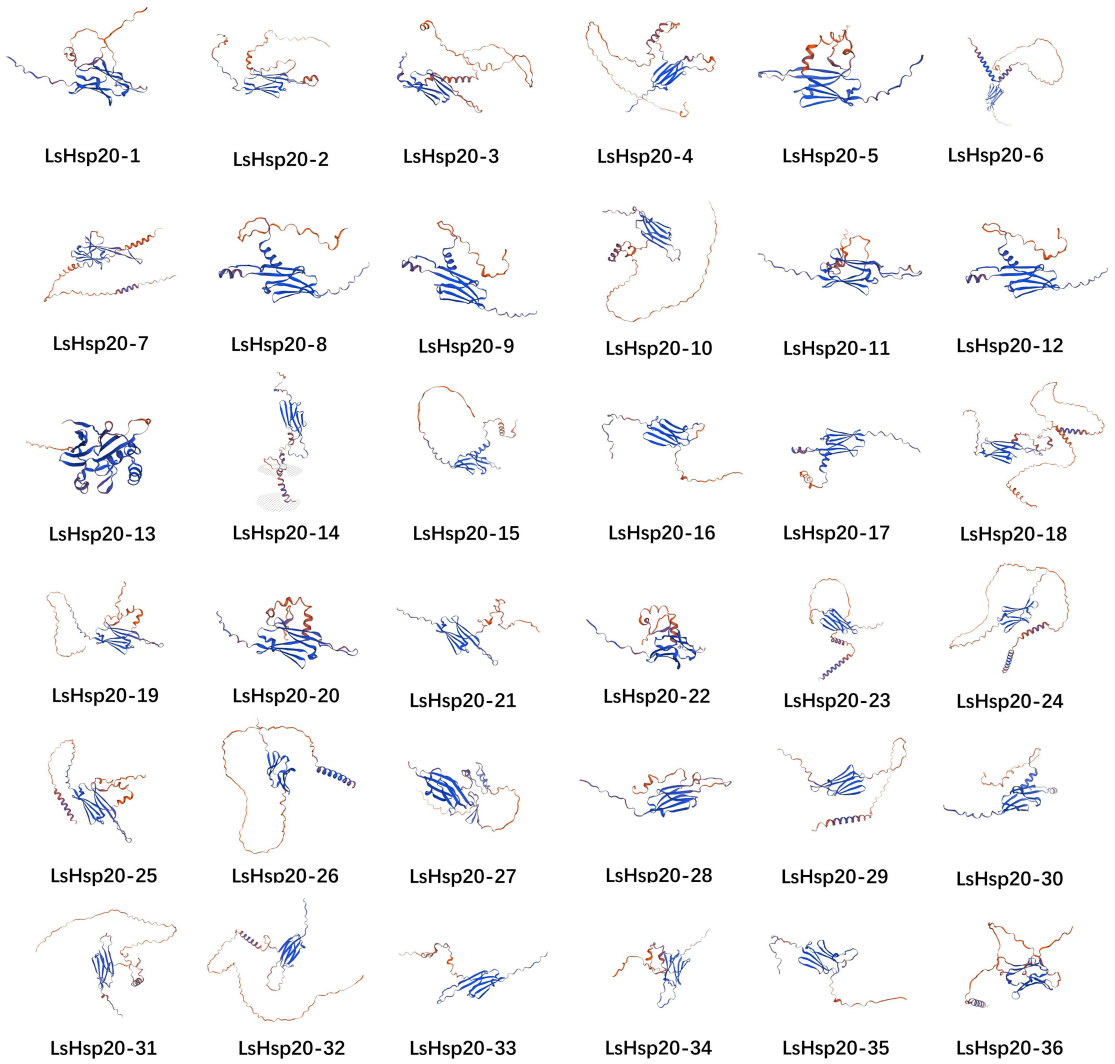
The tertiary structure of proteins of the LsHsp20 family. Using the protein homology modeling method based on the structures of LsHsp20 proteins in the SWISS-MODEL database, the structure with the highest score was chosen as the optimal structure for the LsHsp20 proteins.

An analysis of the secondary structures of the lettuce LsHsp20 proteins is shown in **Supplementary Table S5**. The secondary structures of the 36 proteins were composed of alpha helices, extended strands, random coils, and beta turns but were predominantly composed of random coils. The secondary structure proportion for the LsHsp20 protein family was as follows: random coil > alpha helix > extended strand > beta turn. Among them, alpha helices accounted for 10.68%~38.65%, extended strands accounted for 13.95%~25.76%, random coils accounted for 34.97%~60.52%, and beta turns accounted for 3.4%~9.31%. To determine the reasonable theoretical structures of LsHsp20 proteins, the three-dimensional structures of 36 lettuce LsHsp20 family members were predicted using the SWISS-MODEL homology modeling method (**Figure 2**), and the structure with the highest coverage score was selected as the best structure of the LsHsp20 proteins. The results showed that all 36 LsHsp20 proteins were oligonucleotides and contained α folds, and 44% of the LsHsp20 protein models had no similarity.

### 3.4 Chromosomal location, collinearity analysis, and evolutionary selection pressure analysis of the lettuce *LsHsp20* genes

**Figure 3:**
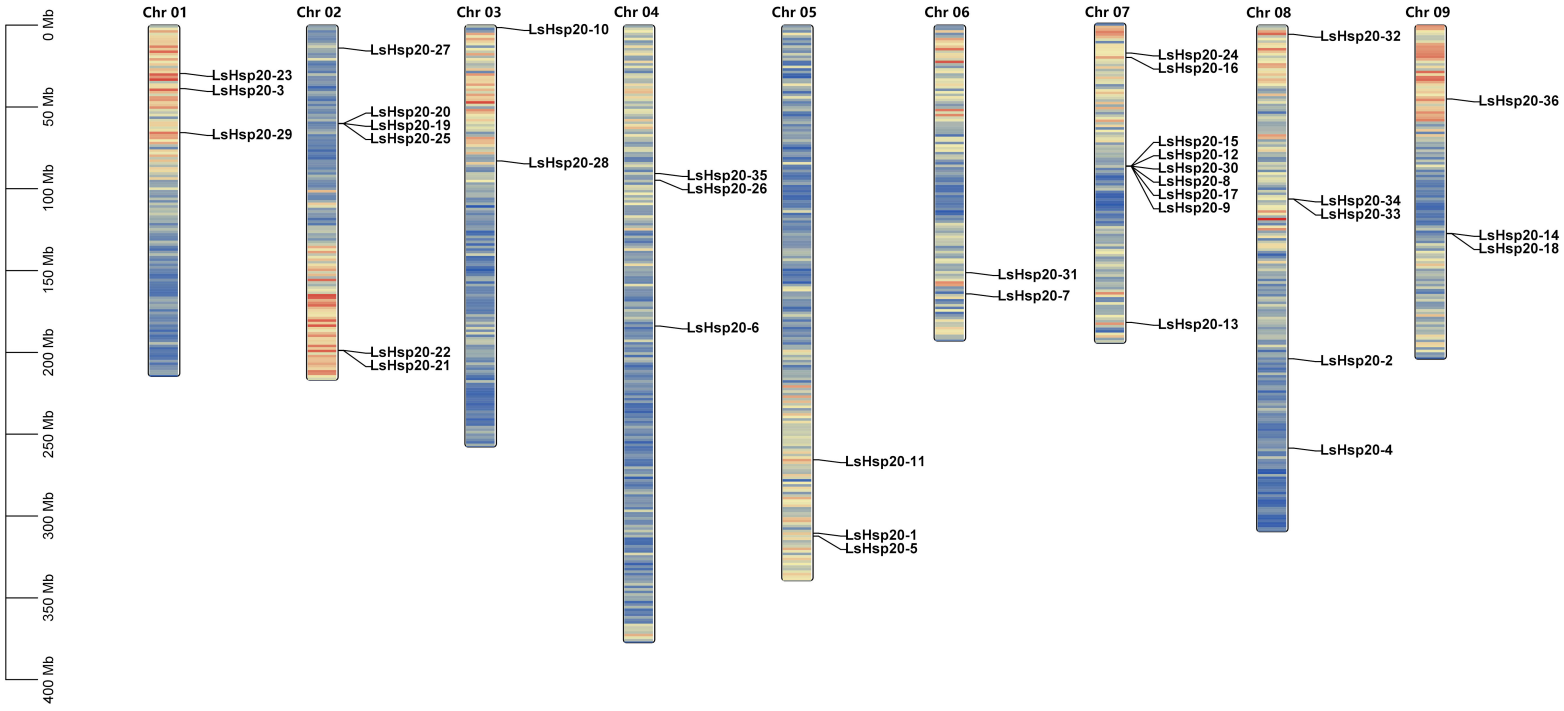
Chromosomal location of the LsHsp20 gene family in lettuce.

**Figure 4:**
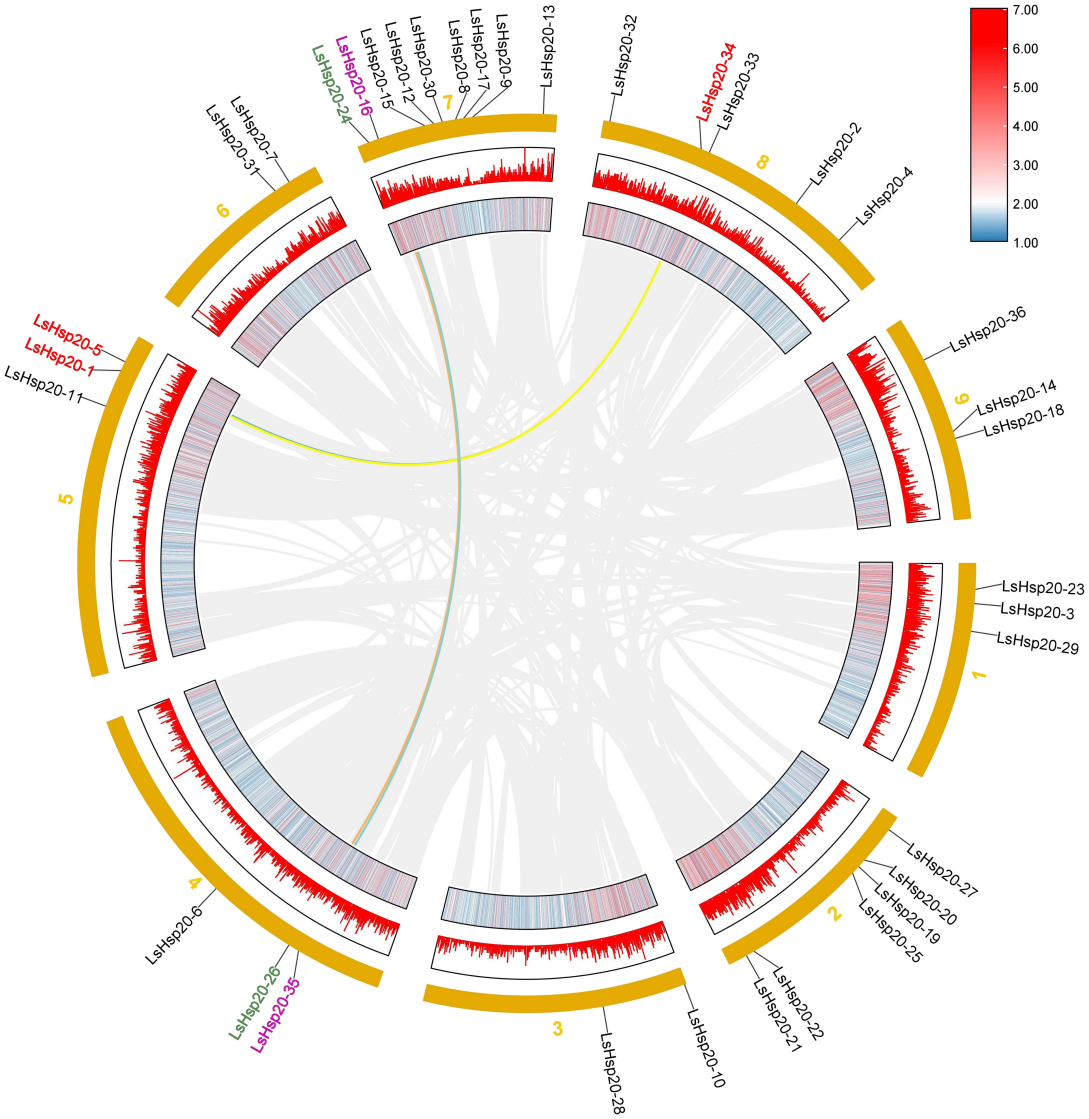
Collinearity analysis of lettuce.

**Figure 5:**
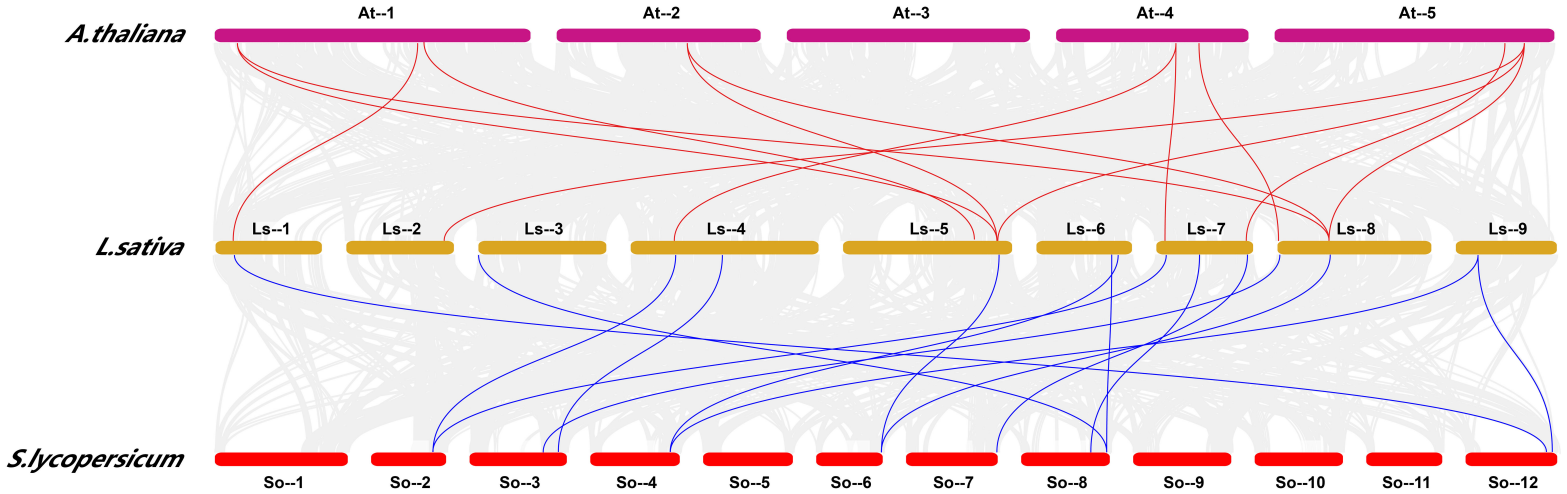
Analysis of the collinearity analysis of lettuce with *Arabidopsis* and tomato plants.

In this study, we further analyzed the chromosomal locations of 36 *LsHsp20* genes in lettuce. As shown in **Figure 3**, these genes were unevenly distributed on nine chromosomes. Chr 07 had up to nine genes, accounting for 25% of the *LsHsp20* genes in lettuce. Chr 02 had six genes; Chr 01, Chr 04, Chr 05, and Chr 09 each had three genes; Chr 02 had four genes; and Chr 03 and Chr 06 had at least two genes each. A total of five gene clusters were found in Chr 02, 07, 08, and 09. Furthermore, we analyzed the duplication events of the *LsHsp20* genes in lettuce (**Figure 4**). Chromosomal evolution and gene replication events inside lettuce were analyzed using collinearity within lettuce species. Four pairs of genes, namely, *LsHsp20–5* and *LsHsp20–34*, *LsHsp20–1* and *LsHsp20–34*, *LsHsp20–16* and *LsHsp20–35*, and *LsHsp20–24* and *LsHsp20–26*, had a collinear relationship and were segmented duplications, and the TBtools software was used to calculate the non-synonymous replacement rate (Ka) and the synonymous replacement rate (Ks). A Ka/Ks ratio greater than, equal to, or less than 1 represents positive, neutral, and purified options, respectively. The Ka/Ks ratios of the four pairs of collinear *LsHsp20* genes in lettuce were less than 1, indicating that the *LsHsp20* genes were mainly purified during *L. sativa* evolution (**Table 2**). To explore the evolutionary relationship of the LsHsp20 family in lettuce, a collinear map of lettuce, *Arabidopsis*, and tomato plant was constructed. The results showed that 13 pairs of genes were collinear in lettuce and *Arabidopsis* (**Figure 5**), 14 pairs of genes were collinear in lettuce and tomato, and 7 pairs of genes were collinear in lettuce, *Arabidopsis*, and tomato.

**Table 2:** Evolutionary selection pressure analysis of *LsHsp20*.

Duplicated gene pairs	Ka	Ks	Ka/Ks	Purifying selection
*LsHsp20–35/LsHsp20–16*	0.12	1.03	0.16	Yes
*LsHsp20–26*/*LsHsp20–24*	0.30	1.47	0.20	Yes
*LsHsp20–1*/*LsHsp20–34*	0.04	1.61	0.03	Yes
*LsHsp20–5*/*LsHsp20–34*	0.20	2.37	0.08	Yes

### 3.5 Phylogenetic analysis of the LsHsp20 protein family in lettuce

**Figure 6:**
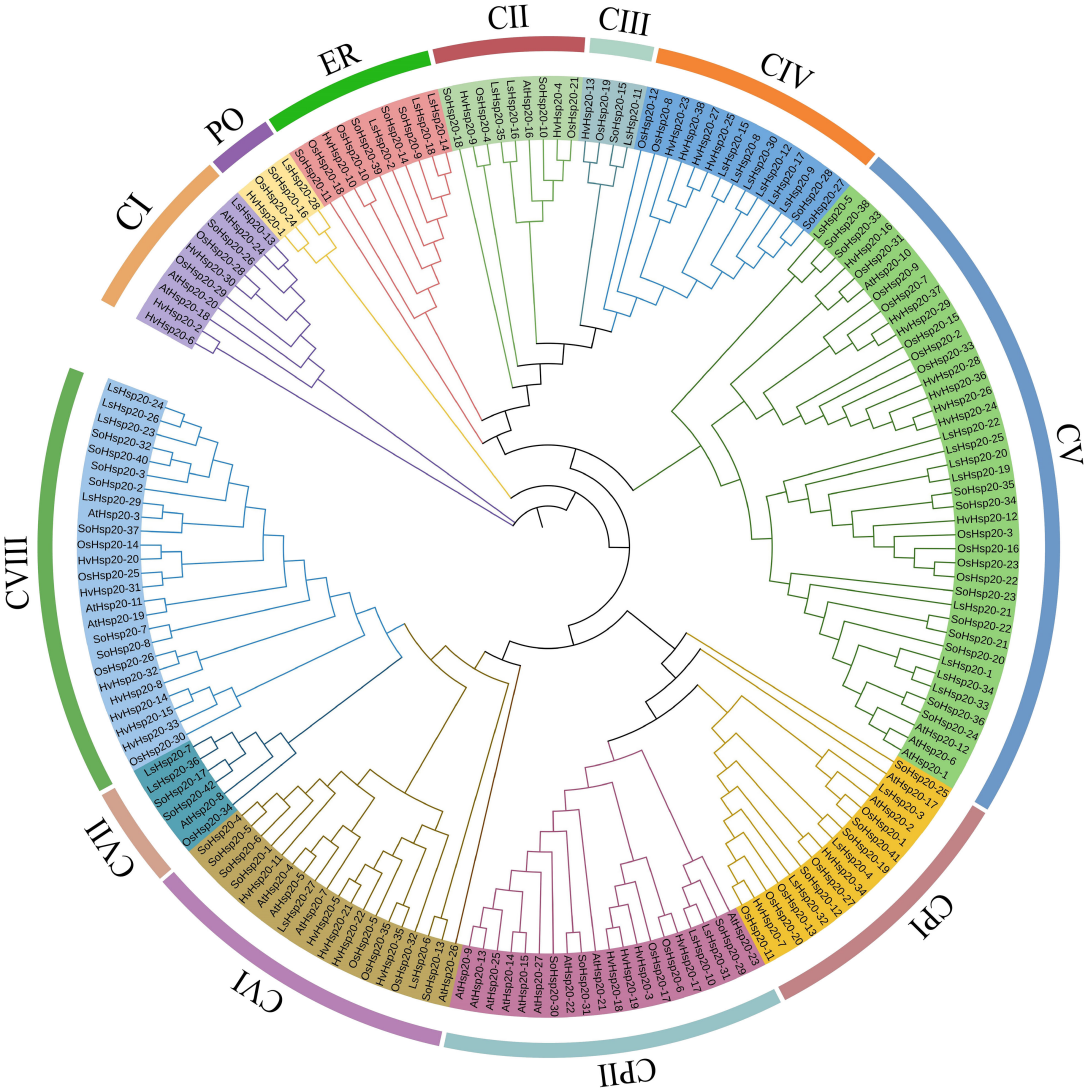
Phylogenetic tree analysis of the LsHsp20 protein family in lettuce and other plants. Ls, *Lactuca sativa*; At, *Arabidopsis thaliana*; Os, *Oryza sativa*; So, *Solanum lycopersicum*; Hv, *Hordeum vulgare*. CI–CVIII indicate that the protein is located in the cytoplasm or nucleus, PO indicates that the protein is located in the peroxisome, CP indicates that the protein is located in the chloroplast, and ER indicates that the protein is located in the endoplasmic reticulum.

To clarify the evolutionary relationship of the Hsp20 family, the sequences of lettuce and other species were compared to construct a phylogenetic tree. MEGA7.0 software was used to construct a phylogenetic tree (**Figure 6**). A total of 178 Hsp20 sequences, consisting of 27 *Arabidopsis thaliana* sequences, 42 tomato sequences, 35 rice sequences, 38 barley sequences, and 36 lettuce sequences, were used in the phylogenetic analysis (**Supplementary Table S6**). According to phylogenetic analysis, the lettuce LsHsp20 proteins can be divided into 12 subgroups (CI, CII, CIII, CIV, CV, CVI, CVII, CVIII, PO, CPI, CPII, and ER), among which 26 (72.2%) of the 36 LsHsp20 proteins belong to the CI–CVIII subgroups.

### 3.6 Analysis of cis-acting elements in the promoters of the lettuce LsHsp20 gene family

**Figure 7:**
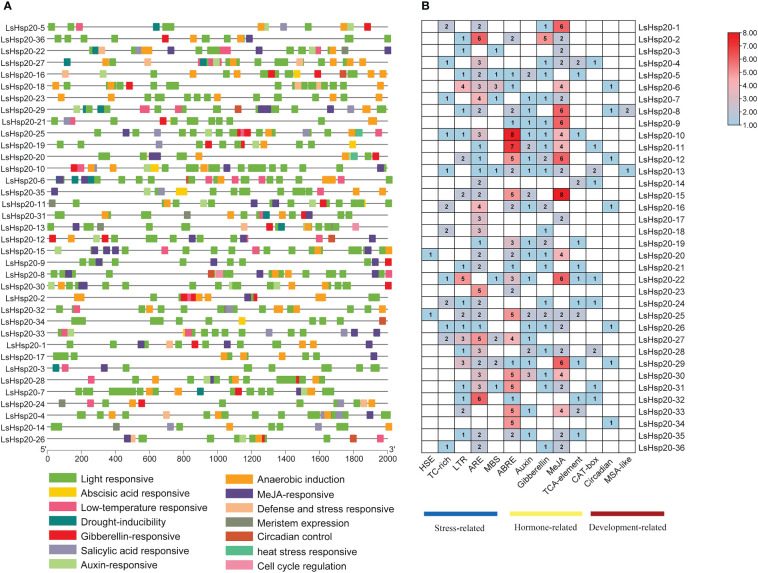
Prediction of the promoter cis-acting elements of the LsHsp20 gene family members in castor. Position **(A)** and number **(B)** of promoter cis-acting elements. HSE, heat stress-responsive cis-acting element; TC-rich, defense and stress signal response elements; LTR, low-temperature signal response element; ARE, anaerobic inducible element; MBS, drought induction element; ABRE, abscisic acid response cis-element; Auxin, auxin response cis-element; Gibberellin, gibberellin response cis-element; MeJA, jasmonic acid response cis-element; TCA-element, salicylic acid response cis-element; CAT-box, meristem expression cis-element; Circadian, circadian rhythm control cis-element; MSA-like, cell cycle regulation cis-element.

The 2,000-bp genomic sequence upstream of the *LsHsp20* genes was extracted to analyze the location and number of cis-acting elements. The 36 genes mainly included three categories: biotic and abiotic stresses, plant hormone responses, and plant growth and development. Five, five, and three cis-acting elements were identified, respectively (**Supplementary Table S7**). Analysis of the number of cis-acting elements in the LsHsp20 family in lettuce revealed that *LsHsp20–10* is the member with the largest number of cis-acting elements in its promoter, of which hormone response elements account for 78.95% and stress response-related elements account for 21.05%. *LsHsp20–3* is the member with the least number of cis-acting elements in the promoter, of which 50% are biotic and abiotic stress elements and 50% are plant hormone response elements. All cis-elements of *LsHsp20–9* are involved in the hormone response (**Figure 7**).

### 3.7 Expression patterns of the *LsHsp20* genes under drought stress

**Figure 8:**
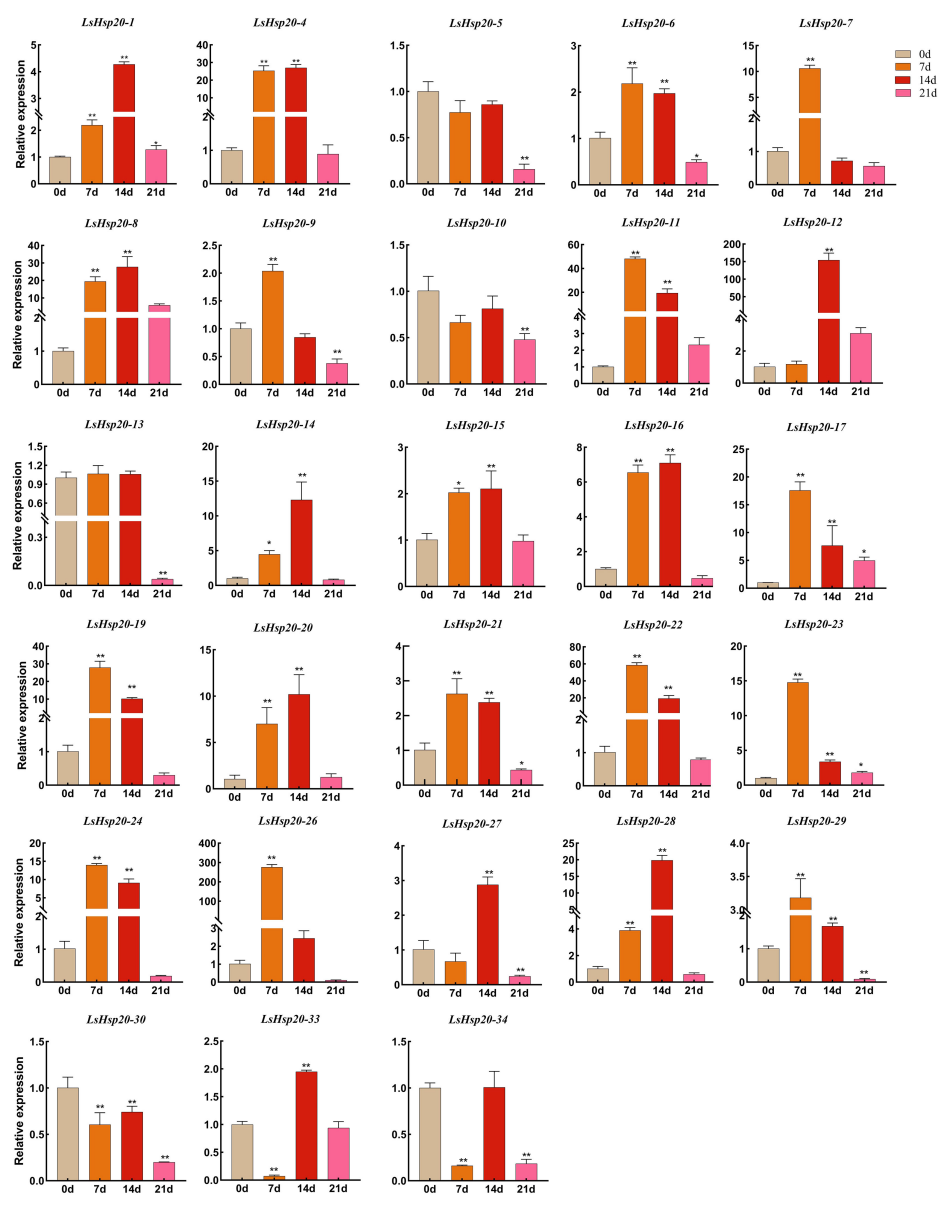
Expression levels of the *LsHsp20* genes under drought stress. All data shown are the means SD of three biological replicates. The asterisks indicate the level of significance (*p < 0.05, **p < 0.01) based on Duncan’s multiple range test.

To study the response of LsHsp20 to drought stress, qRT-PCR was used to analyze the expression of LsHsp20 after drought treatment. There were differences in the relative expression levels of 28 *LsHsp20* genes under drought stress (**Figure 8**). The relative expression of *LsHsp20* exhibited fluctuations over the course of a 21-day drought treatment. Except for the *LsHsp20–5*, *LsHsp20–10*, *LsHsp20–13*, *LsHsp20–30*, and *LsHsp20–34* genes, the expression levels of other *LsHsp20* genes were significantly increased on the 7th and 14th days of drought treatment, and the expression levels of most genes were 2–60 times of the normal level. Notably, the expression of *LsHsp20–12* and *LsHsp20–26* genes was 153 and 273 times the normal level, respectively. The expression of most *LsHsp20* genes was downregulated on day 21 of drought treatment or had no significant difference compared with day 0. The expression levels of *LsHsp20–5*, *LsHsp20–10*, and *LsHsp20–30* were downregulated in each drought treatment period. In general, most *LsHsp20* genes are responsive to drought stress.

## 4 Discussion

Hsp20 proteins have the largest number of members in plants. They are not only involved in the regulation of plant growth and development at specific developmental stages but also inhibit the irreversible aggregation of denatured proteins through molecular chaperone mechanisms when plants are subjected to abiotic stress, thereby enhancing plant resistance to adverse stress (Neto et al., 2020). To date, the Hsp20 gene family has been identified in multiple dicot and monocot species, such as rice (Ouyang et al., 2009), barley (Li and Liu, 2019), apple (Yao et al., 2020), peach (Lian et al., 2022), and pumpkin (Hu et al., 2021). However, no comprehensive study or identification of the lettuce Hsp20 family has been done.

The number of *Hsp20* genes may correlate with the different genome sizes of different plant species or to the expansion or reduction in the number of genes caused by genome duplication or loss during plant evolution (Guo et al., 2015; Qi et al., 2022). In this study, 36 *Hsp20* genes were identified in lettuce, which was less than those in potato (48 genes) (Zhao et al., 2018) and tomato (42 genes) (Yu et al., 2016) and more than in *Arabidopsis* (19 genes) (Siddique et al., 2008) and *Coix* (32 genes) (Hua et al., 2023). According to previous studies, a 200-kb chromosomal region containing two or more genes located on the same chromosome is defined as a tandem duplication (Ma et al., 2016). This study revealed that 36 *LsHsp20* genes were unevenly distributed on nine lettuce chromosomes. We identified five gene clusters, each containing at least two genes. In addition, in chromosome 7, many LsHsp20 family members clustered together (**Figure 3**). In the duplication event, we found that four pairs of genes had segmental duplications (**Figure 5**). These phenomena indicate that tandem duplication and segmental gene duplication events may have increased the number of LsHsp20 gene family members. This result is similar to the results obtained for pigeon pea and bread wheat (Muthusamy et al., 2017; Ramakrishna et al., 2022). To better understand the relationships between the *LsHsp20* genes and *Hsp20* genes from other species, we conducted a species collinearity analysis on lettuce, *Arabidopsis*, and tomato. Thirteen pairs of genes were collinear between lettuce and *Arabidopsis*, and 14 pairs of genes were collinear between lettuce and tomato, indicating that more than half of the *LsHsp20* genes had no collinear relationship with *Arabidopsis* and tomato, which indicates that the lettuce *LsHsp20* genes were relatively conserved during evolution (Ji et al., 2019; Gong et al., 2021; Qi et al., 2022).

Gene structure plays a very important role in plants and facilitates a better understanding of the evolution of gene families in species (Silva et al., 2022). Studies have shown that when plants are subjected to biotic or abiotic stress, genes with fewer or no introns can be quickly activated to help plants cope with the stress, and genes with fewer or no introns have higher expression levels in plants (Ren et al., 2006; Wang et al., 2021). The gene structure analysis of the 36 identified lettuce LsHsp20 gene family members showed that 15 genes (41.7%) had no introns and 20 genes (55.6%) had one intron. *LsHsp20–27* has two introns, the LsHsp20 gene family shows similar motif arrangement in each phylogenetic subgroup, and genes in the same subgroup have the same intron phase, which indicates that the structure may be relatively conservative during evolution, which is similar to the finding of previous studies on apple and *Dendrobium catenatum* (Sarkar et al., 2009; Yao et al., 2020; Wang et al., 2024). The 36 LsHsp20 proteins were divided into 12 subfamilies, 26 of which were located in the cytoplasm and nucleus (CI, CII, CIII, CIV, CV, CVI, CVII, and CVIII), constituting the largest subgroup branch, indicating that the cytoplasm and nucleus may be the main functional sites of the LsHsp20 family. This phenomenon has also been confirmed in other species, such as tomato (Yu et al., 2016) and *Cannabis sativa* (Huai et al., 2022). Studies have shown that plants may have lost or reacquired new genes during the evolution. This study did not identify subgroups such as CX, CXI, CIX, MI, MII, and P from the lettuce LsHsp20 proteins. Similar findings have been reported for other species. In pumpkin, the Hsp20 family lacks subfamilies such as CVI, CVI, and CVIII (Hu et al., 2021). In cucumber, the Hsp20 family lacks CIII, CX, CXI, and other subfamilies (Huang et al., 2022). The loss of certain genes in the lettuce LsHp20 family during evolution may have led to a lack of subgroups.

Studies have shown that promoter cis-elements play important roles in plant physiological responses to biotic and abiotic stresses (Lopes-Caitar et al., 2013; He et al., 2018; Sun et al., 2022). Three major categories of response elements were identified in the promoter regions of the *Hsp20* genes, including hormone response elements, stress response elements, and plant development-related elements (**Figure 7**), among which hormone response elements accounted for the greatest proportion. These findings indicate that the lettuce *Hsp20* genes have multiple or specific functions. Studies have shown that plant hormones can participate in the regulation of plant growth and development and can finely regulate environmental stress through interactions between different hormone signaling pathways (Sato et al., 2018). *AtHSP17.8* is involved in regulating ABA-mediated signaling by overexpressing genes in *Arabidopsis* and lettuce, resulting in a resistance phenotype when plants face environmental stress (Kim et al., 2013). Overexpression of *MsHsp16.9* increased ABA biosynthesis and accumulation in plants, suggesting that *MsHsp16.9* may act as a positive regulator of ABA signaling in *Arabidopsis* (Yang et al., 2017).

Most Hsp20 can be strongly induced under abiotic and biotic stress conditions, including high temperature, drought, salinity, low temperature, heavy metals, hypoxia, and some pathogenic bacteria, thereby enhancing plant tolerance (Peng et al., 2023; Jung et al., 2024). Under high-temperature stress in apples, the expression of 12 *Hsp20* significantly increased more than 1,000-fold after 4 h of heat stress (Yao et al., 2020). In potato, the expression of most *StHsp20* genes was upregulated under high temperatures, drought, and salt stress (Zhao et al., 2018). The overexpression of three *HvHsp20* genes in barley can improve plant resistance to heat stress and biotic stress (powdery mildew) (Li and Liu, 2019). In our study, under drought stress, the expression levels of 23 *LsHsp20* genes were significantly upregulated, among which the expression levels of *LsHsp20–12* and *LsHsp20–16* significantly increased by 153- and 273-fold on the 14th and 7th days of drought treatment, respectively, indicating that the *LsHsp20* genes of lettuce respond to drought stress. These two genes may be more responsive to drought stress and can be used as candidate genes for the selection of drought-tolerant lettuce varieties and their genetic improvement. This study detected the gene expression levels of LsHsp20 in lettuce under drought conditions and found that the gene expression levels of most LsHsp20 increased after drought stress.

## 5 Conclusion

In summary, we performed a genome-wide analysis of the LsHsp20 family in lettuce and identified 36 *LsHsp20* genes. These 36 *LsHsp20* genes are unevenly distributed across nine chromosomes, and the 36 LsHsp20 proteins are divided into 12 subfamilies based on phylogenetic tree and subcellular localization data. To better explore the evolutionary relationships among members of the LsHsp20 family, we analyzed the protein structure, gene structure, conserved motifs, cis-acting elements, and homology between lettuce and other species. The qRT-PCR data revealed significant upregulation of the expression levels of 23 *LsHsp20* genes on the 7th or 14th day of drought stress, indicating strong responsiveness of most LsHsp20 genes in lettuce to drought stress. This indicates that *LsHsp20* genes play an important role in the drought tolerance of lettuce. This study provides information on *LsHsp20* genes and a theoretical basis for the selection of drought-tolerant lettuce varieties and their genetic improvement.

## Data availability statement

The datasets presented in this study can be found in online repositories. The names of the repository/repositories and accession number(s) can be found in the article/**Supplementary Material**.

## Author contributions

QZ: Writing – original draft, Software. BD: Writing – original draft, Formal analysis. MF: Writing – review & editing, Formal analysis. LY: Writing – review & editing, Methodology. CL: Writing – review & editing, Methodology. GH: Writing – review & editing, Data curation. XW: Writing – review & editing, Data curation. HG: Writing – review & editing, Conceptualization. JL: Writing – review & editing, Conceptualization.
